# Hypoxia Contributes to Poor Prognosis in Primary IDH-wt GBM by Inducing Tumor Cells MES-Like Transformation Trend and Inhibiting Immune Cells Activity

**DOI:** 10.3389/fonc.2021.782043

**Published:** 2021-12-08

**Authors:** Zujian Xiong, Hongwei Liu, Chenqi He, Xuejun Li

**Affiliations:** ^1^ Department of Neurosurgery, Xiangya Hospital, Central South University, Changsha, China; ^2^ Xiangya School of Medicine, Central South University, Changsha, China; ^3^ Hunan International Scientific and Technological Cooperation Base of Brain Tumor Research, Xiangya Hospital, Central South University, Changsha, China

**Keywords:** primary IDH-wt GBM, single-cell analysis, hypoxia, MES-like transformation, tumor immune microenvironment

## Abstract

**Aims:**

To reveal the influence of hypoxia on tumor cells and immune cells in primary IDH-wt glioblastoma patients.

**Methods:**

Single-cell RNA-seq data and bulk RNA-seq data were acquired from the Gene Expression Omnibus (GEO) and The Cancer Genome Atlas (TCGA) databases, respectively. Hypoxia status and subtypes of tumor cells were identified based on single-sample Gene Set Enrichment Analysis (ssGSEA). Regulon network analysis of different subtypes under different conditions was conducted by SCENIC. Within tumor microenvironment, biological process activity analysis and cell–cell communication network were conducted to uncover the inner links between each cell subtype under different hypoxia status.

**Results:**

Different types of tumor cell in GBM possessed different hypoxia status, and MES-like subtype was under a more severe hypoxia condition than other subtypes. Hypoxia also induced MES-like signature gene expression within each tumor cell, which could stimulate tumor cell proliferation and invasion by regulating cell–cell communication. Additionally, hypoxia inhibited immune cell activity in the tumor microenvironment by inducing macrophage phenotype polarization and upregulating immune-inhibited cell–cell interaction within immune cells. Interactions between tumor cells and immune cells under hypoxia status also promoted tumor progression.

**Conclusions:**

Hypoxia was a poor prognostic marker for primary IDH-wt GBM patients. Meanwhile, it could induce tumor cells’ MES-like transformation trend and inhibit antitumor function of immune cells.

## Introduction

Glioblastoma (GBM) is the most fatal tumor in the central nervous system (CNS) with the median overall survival of 12–15 months. A previous study has divided GBM into four subtypes, namely, mesenchymal (ME), classical (CL), proneural (PN), and neural (NE), based on bulk RNA-seq data (1) and later retain three subtypes including ME, CL, and PN (2). Among these subtypes, ME and CL are associated with poor prognosis compared with others (3). To evaluate the intratumoral heterogeneity, a recent study identified four cellular subtypes in GBM based on single-cell data, which are a bit different from the former Verhaak’s classification (4). It distinguishes GBM cells into various states named neural-progenitor-like (NPC-like), oligodendrocyte-progenitor-like (OPC-like), astrocyte-like (AC-like), and mesenchymal-like (MES-like) based on cell meta modules, which can coexist within the same tumor sample because of intratumoral heterogeneity. Among all tumor cell subtypes, MES-like subtype is linked to hypoxia, indicating the inner association between MES-like subtype and hypoxia.

Hypoxia, a microenvironment feature in solid tumor, is associated with many cancer “hallmarks,” involving impaired immune response, metabolic reprogramming, stimulation of tumor angiogenesis, and promotion of tumor invasion (5). It has been shown to be a factor of chemotherapy and radiation resistance in GBM (6), which is also linked to a proneural–mesenchymal transformation (PMT) in the transcriptome (7). Moreover, hypoxia is a strong inducer of PMT in GBM by upregulating specific transcription factors (TFs) like hypoxia-inducible factor 1-alpha (HIF1α) (8). In the process of PMT, MES-related gene signatures are associated with therapeutic resistance, while PN-related signatures are associated with therapeutic sensitivity (7). Several lines of evidence suggest that PMT occurs in glioma stem cells under hypoxia status (9), then promotes tumor invasion, angiogenesis, and therapeutic resistance.

Collectively, the vast majority of studies focusing on hypoxia effect are based on bulk RNA-seq datasets. Due to intratumoral heterogeneity, tumor purity, and single-cell sequencing development, hypoxia evaluation through bulk data is unable to reveal specific hypoxia status of each cell type, which, however, can be complemented by single-cell analysis. To study the influence of hypoxia on single cells, including tumor and non-tumor cells, especially immune cells in the tumor microenvironment, we performed the study on single-cell RNA-seq datasets derived from primary IDH-wild type (IDH-wt) GBM patients. This work provided an opportunity to investigate the influence of hypoxia on single cells and uncover new possible mechanisms for hypoxia as a poor prognostic marker.

## Materials and Methods

### Datasets and Data Processing

**Figure 1:**
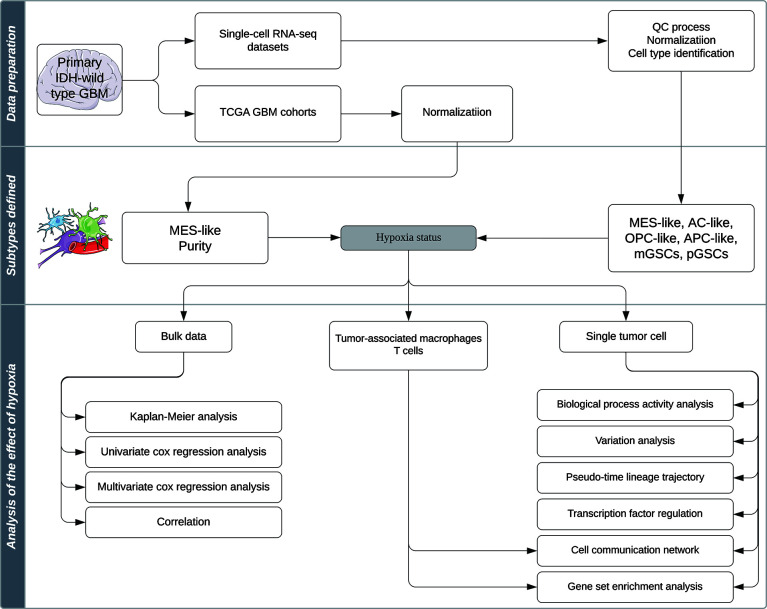
Schematic representation of the analysis pipeline in this study.

A total of 7,930 cells from primary IDH-wt GBM patients’ single-cell RNA-seq data according to Smart-seq2 protocol (GSE131928) were downloaded from the GEO database (https://www.ncbi.nlm.nih.gov/geo) (4). We additionally downloaded 4,825 cells from oligodendroglioma patients’ and 6,341 cells from IDH-mutant astrocytoma patients’ single-cell RNA-seq data according to Smart-seq2 protocol (GSE70630, GSE89567) to test the efficiency of the biological process activity (BPA) transformation analysis (10–12). Six GBM primary cell lines RNA-seq data (three samples in normoxia and three samples in hypoxia) were downloaded from GSE108013 (13). One hundred forty-five samples of Illumina HiSeq RNA-seq data of primary IDH-wild-type GBM and corresponding phenotype data were downloaded from the TCGA database (https://tcga-data.nci.nih.gov/) *via* Xena Browser developed by University of California Santa Cruz (UCSC). The QC process, normalization, and cell-type identification of single-cell RNA-seq data were performed as described in published studies (4, 10, 11), which used the same criteria. RNA-Seq data of TCGA and GSE108013 were normalized by transcripts per kilobase million (TPM) method for further analysis. A computational pipeline for analyzing effect of hypoxia in this study is exhibited in **Figure 1**.

### Subtypes and Glioma Stem-Like Cells (GSCs) Identification

Subtypes of GBM cells and glioma stem-like cells (GSCs) were identified by ssgsea.GBM.classification R package (2). First, we generated numerous virtual samples by randomly selecting expression values of the gene as virtual samples’ corresponding gene expression from datasets. Then, the ssGSEA scores of each category were calculated, respectively. We set 5,000 virtual random samples and correlated these samples with the real sample. At the same time, we counted the number of matches with random samples under each subtype. We defined the subtype as the one that had the fewest matches to the random sample. If there were more than one subtypes sharing the min matches in one sample, we defined the sample as MIX. Besides, we defined the cells with no random sample match as mesenchymal GSCs (mGSCs) or proneural GSCs (pGSCs). The four GBM subtypes (MES-like, AC-like, OPC-like, and APC-like) signatures were acquired from previous research (4). Additionally, mGSCs and pGSCs signatures were obtained from Wang’s study (14). Meanwhile, macrophage-subtype marker genes were obtained from the CellMarker database (15).

### Hypoxia Score Calculation and Hypoxia Status Identification

According to the hypoxia 15-gene signature evaluated by published papers (16–20) in pan-cancer analysis, we used GSVA algorithm (21) to calculate the hypoxia score in TCGA bulk samples and single-cell samples, respectively. Unsupervised hierarchical clustering method was employed to classify hypoxia status of bulk samples based on 15 hypoxic gene expression profiles, same as the process in previous research (16). The top three clusters were designed as hypoxia, intermediate, and normoxia groups in TCGA GBM cohorts. Due to the dropout rate and sparse matrix of the single-cell data, we used quarter quantile to identify the hypoxia status of cells. The cells possessing the upper quarter hypoxia score were labeled as hypoxia status, and the cells with the lower quarter hypoxia score were identified as normoxia status; the other cells were defined as intermediate status.

### MES-Like Score Calculation and Deconvolution Analysis

We used the same method as calculating hypoxia score to acquire the MES-like score of each GBM tumor cell based on MES-like signatures. The deconvolution of TCGA GBM samples were conducted by CIBERSORTx, in which we got the target cell proportion within the bulk samples (22).

### Tumor Purity Calculation in Bulk Samples and Purity–Hypoxia Correlation

We adopted five tumor purity scores in the analysis, namely, the consensus purity estimation (CPE) score, ESTIMATE score, ABSOLUTE score, LUMP score, and IHC score. ESTIMATE was based on specific gene expression profiles consisting of 141 immune genes and 141 stromal genes (23); ABSOLUTE used somatic copy-number data (24); LUMP (leukocytes unmethylation for purity) averaged 44 non-methylated immune-specific CpG sites; and IHC was estimated by image analysis of hematoxylin and eosin stain slides produced by the Nationwide Children’s Hospital Biospecimen Core Resource (25). For CPE, it was the median purity level derived from the four methods mentioned above after normalization as previously described (25). We used Pearson correlation to calculate the coefficient of purity score and hypoxia score among different calculation methods.

### Pseudo-Time Lineage Trajectory

Lineage trajectory was constructed by monocle R package (26). Briefly, we selected variant features identified by Seurat v3 as an input gene set. Then, the monocle used a machine learning method to order single cell into a trajectory in a two-dimension space.

### Biological Process Activity Analysis and GSEA Enrichment

Biological process activity (BPA) analysis was based on gene list in msigdb database to evaluate each pathway’s activity in single cells (12). We used KEGG database, GO database, and msigdb immune-related C7 database to calculate pathways activity in cells, in which the parameters were set as advised (12, 27). To evaluate the efficiency of the method, we adopted another two single-cell datasets of glioma as formerly mentioned to test it and visualized BPA result by TSNE. Gene set enrichment analysis (GSEA) was performed by clusterProfiler R package based on gene list from msigdb (28).

### Identification of Differentially Expressed Genes (DEGs) and Pathways

Differentially expressed genes (DEGs) and differentially expressed pathways between the hypoxia and normoxia groups were identified by limma R package. All parameters were set as default.

### Transcription Factor Regulation and Cell Communication Network Identification

We used epigenetic landscape *in silico* deletion analysis (LISA), based on DEGs, to identify main transcription factors in hypoxia group (29). To analyze transcription factor regulons further, we adopted SCENIC R package (30) with default parameters. For visualization, we mapped the regulon activity (AUC) scores in heatmap plot. Additionally, intratumoral cell–cell communication network based on potential receptor–ligand interaction was inferred by CellPhoneDB from single-cell transcriptomic data (31).

### Survival and Statistics Analysis

R packages survival and survminer were used for overall survival analysis. Univariate cox and multivariate cox regression analysis methods were conducted to identify independent prognostic features. In addition, single- and two-factor Kaplan–Meier curves were shown to compare the prognostic results between different groups. All statistical analyses were performed using R software, version 3.6.2 (The R Foundation for Statistical Computing, http://www.rproject.org/). Continuous variables that conform to normal distribution between groups were compared by the Student’s t-test with *post-hoc* pairwise Bonferroni tests. Variables that do not conform to the normal distribution were compared by the Wilcoxon rank-sum test with *post-hoc* pairwise Bonferroni tests.

## Results

### Hypoxia Contributes to Poor Prognosis in Primary IDH-wt GBM Bulk Samples

**Figure 2:**
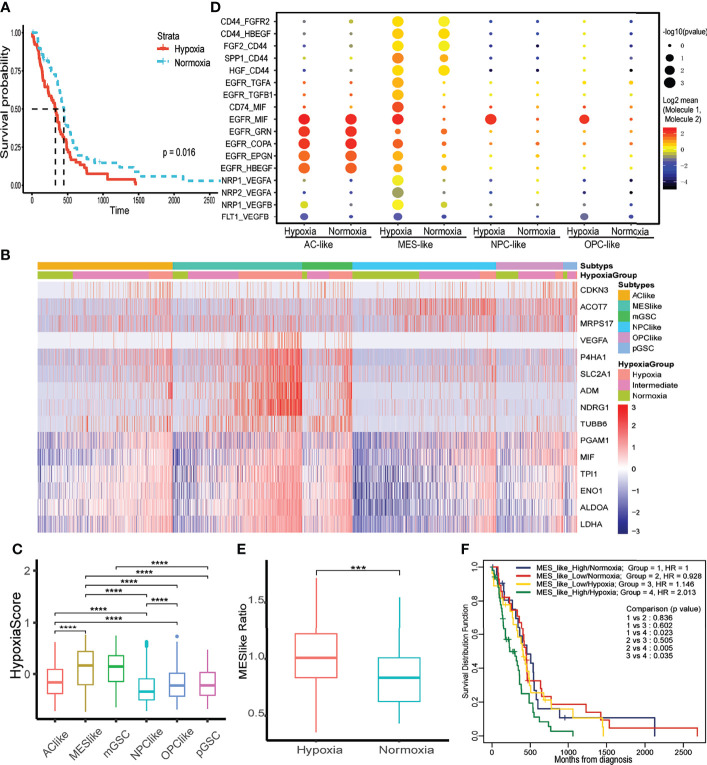
The effect of hypoxia on tumor cells and prognosis. **(A)** Kaplan–Meier curve of different hypoxia status of primary IDH wild-type GBM in TCGA database. **(B)** The expression of 15 hypoxia-related genes in GBM tumor cells. **(C)** The distribution of hypoxia score within each tumor cell type. **(D)** Intercellular communications within each tumor subtype. **(E)** Distribution of MES-like cell proportion within TCGA primary IDH-wt GBM samples between hypoxia and normoxia group. **(F)** Two-factor Kaplan–Meier curve of TCGA primary IDH-wt GBM. The two factors were MES-like cell proportion and hypoxia status of each sample. The character ***, **** means p < 0.001 and p < 0.0001, respectively.

We divided 145 primary IDH-wt GBM patients from TCGA database into three hypoxia status: hypoxia (n = 78), intermediate state (n = 17), and normoxia (n = 50) based on 15-gene hypoxic signature, which performed the best in evaluating tumor hypoxic condition reported by former study (**Supplementary Figure S1A**) (16). To investigate the effect of hypoxia, we took hypoxia and normoxia groups into further analysis, and we observed that hypoxia was associated with worse prognosis (**Figure 2A**). Additionally, we conducted univariate Cox regression on hypoxia status and found that it was a risk factor [hazard ratio (HR) = 1.63; 95%CI = 1.09–2.42, p = 0.016].

### Identify Hypoxia Status in Primary IDH-wt GBM Single Cells

To thoroughly evaluate hypoxia effects on primary IDH-wt GBM, we calculated hypoxia score on six subtypes of tumor cells in GBM (MES-like, AC-like, OPC-like, NPC-like, mGSC, and pGSC). We first identified four subtypes of GBM cells (MES-like, AC-like, OPC-like, and NPC-like) and then classified all tumor cells into another two GSC subtypes. Interestingly, mGSCs were derived from MES- and AC-like subtypes, while pGSCs were derived mainly from OPC- and NPC-like cells (**Supplementary Figure S1B**). Afterwards, we divided each of them into three hypoxia status as previous depicted (**Figure 2B**). These 15 hypoxia marker genes were upregulated in hypoxia groups comparing to intermediate and normoxia groups; meanwhile, we performed LISA analysis based on differentially upregulated genes in the hypoxia group [logFC > 1 and false discovery rate (FDR) < 0.05], discovering that both transcription factors HIF1A and HIF2A (EPAS1) were upregulated in the hypoxia group (**Supplementary Table S1**). We then compared hypoxia score among cell subtypes to distinguish overall hypoxic condition of each subtype (**Figure 2C**). Apart from GSCs, MES-like subtype was the most hypoxic one, followed by AC-, OPC-, then NPC-like subtype. To uncover the relationship between hypoxia status and these four tumor cell subtypes, we analyzed the autocrine signaling under different hypoxia status and found that MES-like subtype with hypoxia sent out more epidermal growth factor receptor (EGFR), vascular endothelial growth factor receptor (VEGFR), and fibroblast growth factor receptor (FGFR) signal to itself, indicating that it was more proliferative and invasive than others (**Figure 2D**). Moreover, we conducted deconvolution analysis on TCGA samples to calculate each subtype proportion by CIBERSORTx and found that hypoxia groups in bulk dataset had more MES-like cells (**Figure 2E**). Consistent with hypoxia, MES-like cell proportion was identified as a risk factor by univariate Cox regression (HR = 1.48, 95%CI = 1.01–2.18, p = 0.046) and possessed synergistic effect with hypoxic condition on poor prognosis (**Figure 2F**). In order to explore potential role of the hypoxia and MES-like cell proportion for patients’ prognosis in detail, we included gender, age, O^6^-methylguanine DNA methyltransferase (MGMT) promoter methylation status, and purity to perform multivariate Cox analysis, which suggested that hypoxia was an independent risk factor (**Table 1**). However, we did not find obvious associations between tumor purity and hypoxia, which indicated that hypoxia was attributed to multiple cell types in the tumor microenvironment (**Supplementary Figure S1C**).

**Table 1:** Statistic of prognostic related factors in TCGA cohort.

Variables	Multivariate Cox regression analysis	
	HR (CI95)	p-value
Gender	1.399 (0.851–2.299)	0.186
Age	1.028 (1.006–1.049)	0.011
Hypoxia	1.943 (1.164–3.242)	0.011
MES-like	0.955 (0.543–1.681)	0.873
MGMT status	0.613 (0.360–1.041)	0.070
Purity	0.127 (0.028–0.569)	0.007

### Hypoxia Changes Immune-Related Metabolism and Induced MES-Like Signature Expression in GBM Tumor Cells

**Figure 3:**
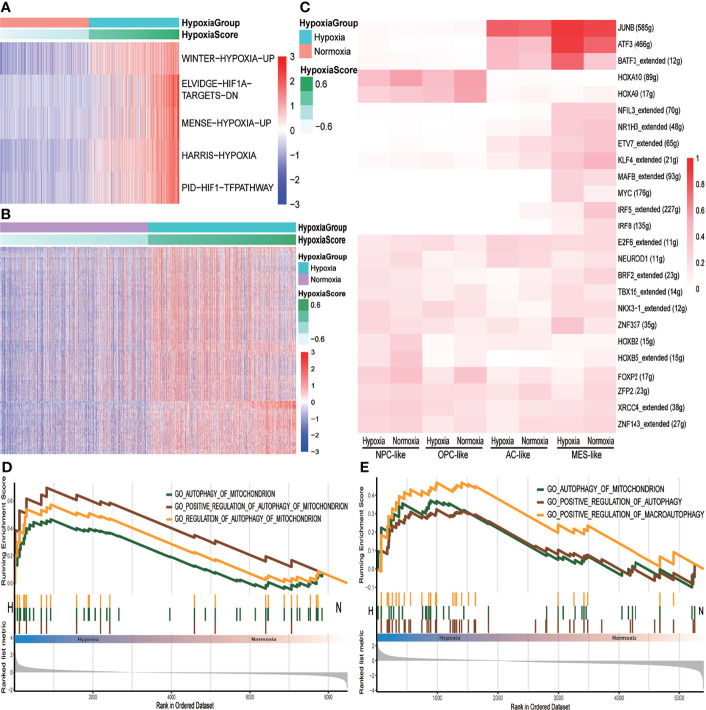
The different pathway activities between hypoxia and normoxia groups. **(A)** Heatmap of KEGG metabolism pathway activity between hypoxia and normoxia groups within tumor cells. **(B)** Heatmap of msigdb C7 immune signatures between hypoxia and normoxia groups within tumor cells. **(C)** Heatmap of binarized regulon network activity of different tumor cell subtype under hypoxia or normoxia. **(D)** GSEA analysis plot of autophagy-related pathways in tumor cells under hypoxia condition with p < 0.05. **(E)** GSEA analysis plot of autophagy-related pathways in MES-like tumor cells under hypoxia condition with p < 0.05.

We performed BPA analysis on primary IDH-wt GBM tumor cells based on KEGG, Msigdb C7, and GO databases. BPA based on single-cell RNA sequencing (scRNA-seq) scRNA-seq data, which could decrease the impact of dropout events and batch effects to provide a robust description on cellular states, had already been tested well in the additional two single-cell datasets (**Supplementary Figure S1D**) (12). We observed that hypoxia- and HIF1A-related pathways in KEGG database were upregulated in the hypoxia group (**Figure 3A** and **Supplementary Table S2**). In addition, there were more immune-related gene sets obviously upregulated in the hypoxia group than in the normoxia group such as interferon (IFN)-, interleukin-15 (IL15)-, and signal transducer and activator of transcription (STAT)-associated signatures, which showed a higher immune activity, whether pro- or anti-tumor, within hypoxia GBM microenvironment (**Figure 3B** and **Supplementary Table S4**). However, few GO pathways were obviously changed between hypoxia and normoxia groups (**Supplementary Table S2**). We then used SCENIC to distinguish regulons between hypoxia and normoxia groups. As a result, ATF3, JUNB, and MYC regulons were highly expressed in the hypoxia group (**Supplementary Figure S2A**). For more details about hypoxia and cell subtypes, we conducted SCENIC again on both cell subtypes and hypoxia status and found that MES-like subtype shared more common regulons with AC-like subtype revealing that these two subtypes were from the same progenitor that differentiated to different directions due to microenvironment (**Supplementary Figure S2B**) (32), while NPC-like subtype shared more common regulons with OPC-like subtype (**Figure 3C**). Within the MES- or AC-like cells, ATF3, a key inhibitory of transcriptional regulator in the inflammatory response, was higher in the hypoxia group. Since autophagy, which could be induced by hypoxia, was a significant catabolic mechanism for GBM tumor cells to survive and resist antitumoral therapy (33), we tested autophagy pathways’ activity between hypoxia and normoxia groups by GSEA. The results showed that these pathways were enriched in the hypoxia group, especially at MES-like subtype, with statistical significance comparing to other subtypes (**Figures 3D, E**).

**Figure 4:**
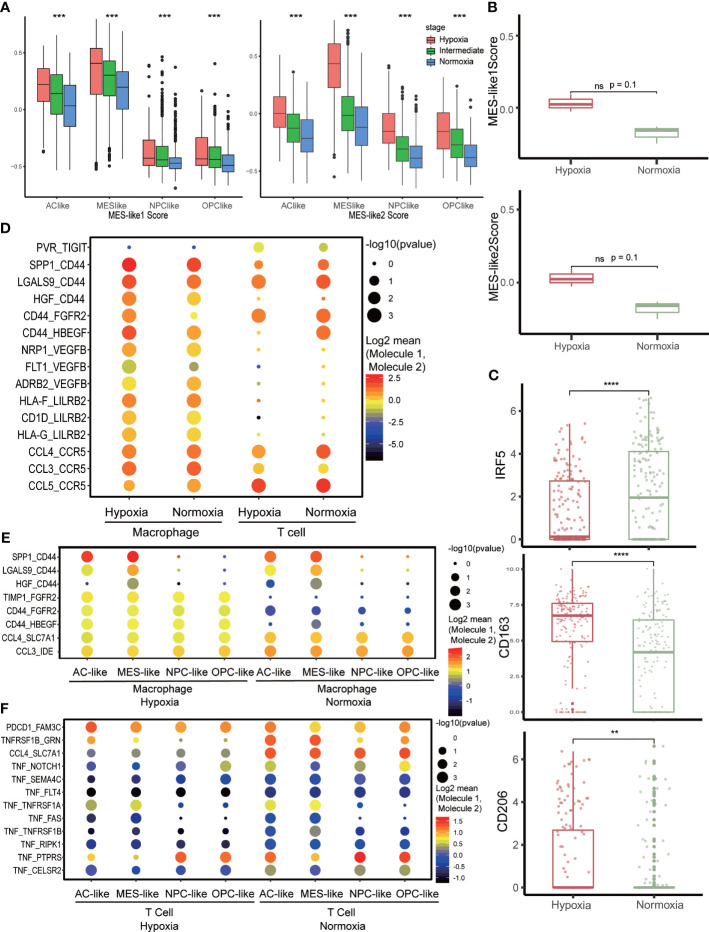
Cell–cell communication network in the tumor microenvironment. **(A)** The distribution of MES-like score in each tumor cell subtype under different hypoxia status. **(B)** The distribution of MES-like score in primary cell lines of primary IDH-wt GBM cultured under different hypoxia conditions. Hypoxia group was cultured under 3% O_2_ for 48 h, and normoxia group was cultured under 21% O_2_ for 48 h. **(C)** The expression of macrophage marker gene in hypoxia or normoxia group. M1 marker, IRF5 and M2 markers, and CD163 and CD206 were selected. **(D)** Intercellular interaction within immune cells under different hypoxia conditions. **(E)** Cell–cell communications between macrophages and tumor cells under different hypoxia conditions. **(F)** Cell–cell communications between T cells and tumor cells under different hypoxia conditions. The character **, ***, **** means p < 0.01, p < 0.001 and p < 0.0001, respectively. ns, no significance.

Meanwhile, we also discovered that CD44, an MES marker gene, was higher in the hypoxia group (**Supplementary Figure S2C**). This was consistent with the former analysis that MES-like cell proportion was higher in the hypoxia group in bulk tissue (**Figure 2E**). We then calculated MES-like score among subtypes of tumor cells based on two identified MES-like signatures (4) and found that with the exacerbation of hypoxia, the MES1- and MES2-like score all increased within each subtype (**Figure 4A**). To verify that hypoxia could induce MES-like signatures expressed in GBM tumor cells, we analyzed another GBM RNA-seq dataset from primary cultured cell lines of primary IDH-wt GBM patients (13). These cells were cultured under hypoxia condition (n = 3, 3% O_2_) or normoxia condition (n = 3, 21% O_2_) for 48 h, respectively. Consistently, two MES-like score of cell lines under hypoxia were higher than normoxia (**Figure 4B**). Because of the limited sample size, we did not observe p-values with statistical significance. However, the possibility of type I error between these two groups was 10%. Considering the limitation of sample size, the error rates were considered acceptable.

### Hypoxia Weaken Antitumor Activity of Immune Cells in GBM Microenvironment

The immune microenvironment of primary IDH-wt GBM mainly consisted of tumor-associated macrophages (TAMs) (34). We selected TAMs identified as hypoxia or normoxia for further analysis (**Supplementary Figure S2D**). The TAMs under hypoxia status highly expressed M2 markers, CD163 and CD206, while TAMs in normoxia upregulated M1 marker, IRF5 (35, 36) (**Figure 4C**). Meanwhile, TAMs were more active in normoxia compare to those in hypoxia (**Supplementary Figures S2E, F**), and CCL-related cell–cell interactions were higher in normoxia status between TAMs and tumor cells (**Figure 4E**). We then tested autocrine signaling within TAMs under different hypoxia status and observed that CD44-related communication, which could induce macrophage M1 to M2 polarization (37), was more frequent and stronger in hypoxia (**Figure 4D**). Furthermore, we investigated T cells with hypoxia and normoxia status as well but found no obvious change in expression of inhibitory immune checkpoint genes (**Supplementary Figure S3A**). However, the TIGIT-related communication was a little stronger within hypoxia T cells (**Figure 4D**), while tumor necrosis factor (TNF)- and FASLG-related communications were stronger in normoxia between T cells and tumor cells (**Figure 4F** and **Supplementary Figure S3B**). Otherwise, tumor cells in hypoxia could inhibit TAMs activation *via* HLA-G/LILRB interactions (**Supplementary Figure S3C**), assist TAMs polarization *via* CD44-related communication (**Figure 4E** and **Supplementary Figure S3C**), and inhibit T-cell activation *via* PDCD1 and TIGIT (**Figure 4F** and **Supplementary Figure S3B**). Conversely, TAMs could promote tumor cells proliferation and invasion *via* EGFR-, FGFR2-, and CD44 (38)-related interaction in hypoxia (**Figure 4E** and **Supplementary Figure S3C**).

## Discussion

The classification of GBM subtypes according to bulk RNA-seq and scRNA-seq possesses inner link as previously reported that TCGA-CL and TCGA-ME subtypes in bulk correspond to tumors enriched with AC- and MES-like cells, respectively, while TCGA-PN subtype corresponds to the combination of OPC- and NPC-like cells (4). TCGA GBM subtypes can also be distinguished by the cell composition that TCGA-CL contain high level of astrocytes, TCGA-ME are infiltrated highly by immune cells, and TCGA-PN are characterized by the highest level of oligodendrocytes and neurons (14). Nowadays, studies on glioma stem cells identify them into two main types, namely, mGSC and pGSC (14, 39). Similarly, the TCGA-ME and TCGA-CL samples are enriched by mGSCs, and TCGA-PN contains high level of pGSCs (14). In our study, we demonstrated that AC-like cells shared more common features with MES-like cells, while OPC-like cells were more similar to NPC-like cells in terms of regulon network and cell–cell communication. Considering all the evidence, we hypothesize that these four GBM cell subtypes come from two lineages derived from mGSC or pGSC, respectively. One is the mesenchymal lineage including mGSC, MES-like subtype, and AC-like subtype, and the other is proneural lineage containing pGSC, OPC-like subtype, and NPC-like subtype. Compared to proneural lineage, the hypoxia degree of mesenchymal lineage was more severe. Such phenomenon is partially associated with metabolism distinction that mGSC preferentially utilizes glycolysis, while pGSC employs oxidative phosphorylation (39). However, the metabolism of each cell subtype of GBM and the lineage hypothesis need further study.

One mechanism of hypoxia inducing GSCs PMT is altering DNA methylation pattern at promoter of mesenchymal-related genes, regulators, or enhancers (7). Nevertheless, we did not find any obvious methylation pattern of mesenchymal markers in methylation data from TCGA (**Supplementary Table S3**). This result can be influenced by tumor purity in that the methylation data contain not only the tumor cells but also non-tumor cells like immune cells and stromal cells. Unfortunately, single-cell methylation analysis on GBM, which would be a meaningful direction for studying hypoxia effect on single cells, does not exist. Preliminary works about PMT of GBM are focused on GSCs, but in our comprehensive analysis, non-GSCs cells of GBM tumor could also upregulate MES-like signature genes under hypoxia. Although hypoxic MES-like cells were more malignant in inner tumor cell interaction such as invasiveness mediated by CD44-related interaction (38), proliferation induced by EGFR- and MIF-related interactions (40), and angiogenesis activated by VEGF-related interaction, other tumor cell subtypes under hypoxia performed more or less similar manners (**Figure 2D**). This MES-like transformation trend under hypoxia environment could partially explain the role of hypoxia as the poor prognosis marker of GBM.

Hypoxia is a microenvironment feature that could influence tumor cells and other non-tumor cells simultaneously. In our results, hypoxia was identified as an independent risk factor for prognosis of GBM patients. Although the result of multivariate Cox regression analysis denied MES-like ratio as a risk factor, it could be explained that hypoxia had inner contact with MES-like ratio, thereby influencing MES-like ratio’s impact on prognosis. In addition, we found that hypoxia could inhibit immune activity. Since the immune microenvironment of primary GBM mainly consisted of TAMs (34), we analyzed TAMs in the datasets and found that TAMs exhibited M2 phenotype in hypoxia status, while they exhibited upregulated M1 markers when in normoxia status. The antitumor ability of macrophages was weakened by hypoxia so that it could benefit tumor growth. Not only cells’ autocrine signaling, like gene expression regulation, hypoxia also influences intercellular interaction between immune cells and tumor cells. In hypoxia status, tumor cells inhibited immune cells’ antitumor ability by interacting with inhibitory receptors in immune cells like PDCD1, TIGIT, and LILRB or weakening the immune-stimulation ligand–receptor interactions such as CCL, TNF, and FASLG. Furthermore, TAMs also promoted tumor progression under hypoxia by interacting with corresponding receptors, such as EGFR- and FGFR2-promoting tumor cell proliferation, CD44-promoting tumor invasiveness, and VEGF-inducing angiogenesis to increase invasion ability of tumor cells.

In summary, we explored in this study the effects of hypoxia on single cells and on both tumor cells and immune cells from primary IDH-wt GBM. Hypoxia was identified as a poor prognostic marker for primary IDH-wt GBM patients by analyzing bulk RNA-seq data. We also explored the potential mechanism of hypoxia contributing to poor prognosis on scRNA-seq data and found that hypoxia could induce tumor cells’ MES-like transformation trend and inhibit immune antitumor function such as inducing macrophage M1 to M2 polarization, thereby promoting tumor progression.

## Data Availability Statement

All data was downloaded from the public database. The single-cell data was got from the GEO database and the bulk data was from the TCGA database.

## Author Contributions

ZX designed the workflow, performed the data analysis, and wrote the manuscript. HL and CH assisted with the data analysis, designed the graphic image, and revised the manuscript. XL assisted with the study design and contributed to the manuscript. All authors contributed to the article and approved the submitted version.

## Conflict of Interest

The authors declare that the research was conducted in the absence of any commercial or financial relationships that could be construed as a potential conflict of interest.

## Publisher’s Note

All claims expressed in this article are solely those of the authors and do not necessarily represent those of their affiliated organizations, or those of the publisher, the editors and the reviewers. Any product that may be evaluated in this article, or claim that may be made by its manufacturer, is not guaranteed or endorsed by the publisher.

## Supplementary Material

Supplementary Figure 1
**(A)** The expression of 15 hypoxia-related genes in TCGA GBM samples. **(B)** The composition ratio of 4 GBM tumor cell subtypes in glioma stem like cells. **(C)** The distribution of tumor purity score under different hypoxia conditions and correlation with hypoxia score. **(D)** TSNE plot based on BPA transformation values.

Supplementary Figure 2
**(A)** Heatmap of binarized regulon network activity in tumor cells with hypoxia or normoxia. **(B)** Lineage trajectory among mGSCs, MES-like cells and AC-like cells. **(C)** Distribution of CD44, MES marker, between hypoxia and normoxia groups. **(D)** The expression of 15 hypoxia-related genes in GBM non-tumor cells. **(E)** GSEA analysis plot of activated pathways in macrophage under normoxia condition. **(F)** GSEA analysis plot of activated pathways in macrophage under hypoxia condition.

Supplementary Figure 3
**(A)** The relative expression level about inhibitory immune checkpoints of T cells under different hypoxia conditions. **(B)** Cell-cell communications between tumor cells and T cells under different hypoxia conditions. **(C)** Cell-cell communications between tumor cells and macrophages under different hypoxia conditions.

Supplementary Table 1 The expression rank of transcription factors in tumor cells under hypoxia condition identified by LISA.

Supplementary Table 2 Differentially expressed BPA pathways between different hypoxia groups, hypoxia v.s. normoxia.

Supplementary Table 3 Differentially methylation condition nearby MES-like signature genes between different hypoxia groups, hypoxia v.s. normoxia.

Supplementary Table 4 Immune-related signatures unregulated in hypoxia group within tumor cells showed in **Figure 3B**.
