# Supplementary figures and images for: Hypoxia Contributes to Poor Prognosis in Primary IDH-wt GBM by Inducing Tumor Cells MES-Like Transformation Trend and Inhibiting Immune Cells Activity
Source: Front Oncol. 2021 Dec 8;11:782043. doi: 10.3389/fonc.2021.782043 (PMC8694101; doi:10.3389/fonc.2021.782043)

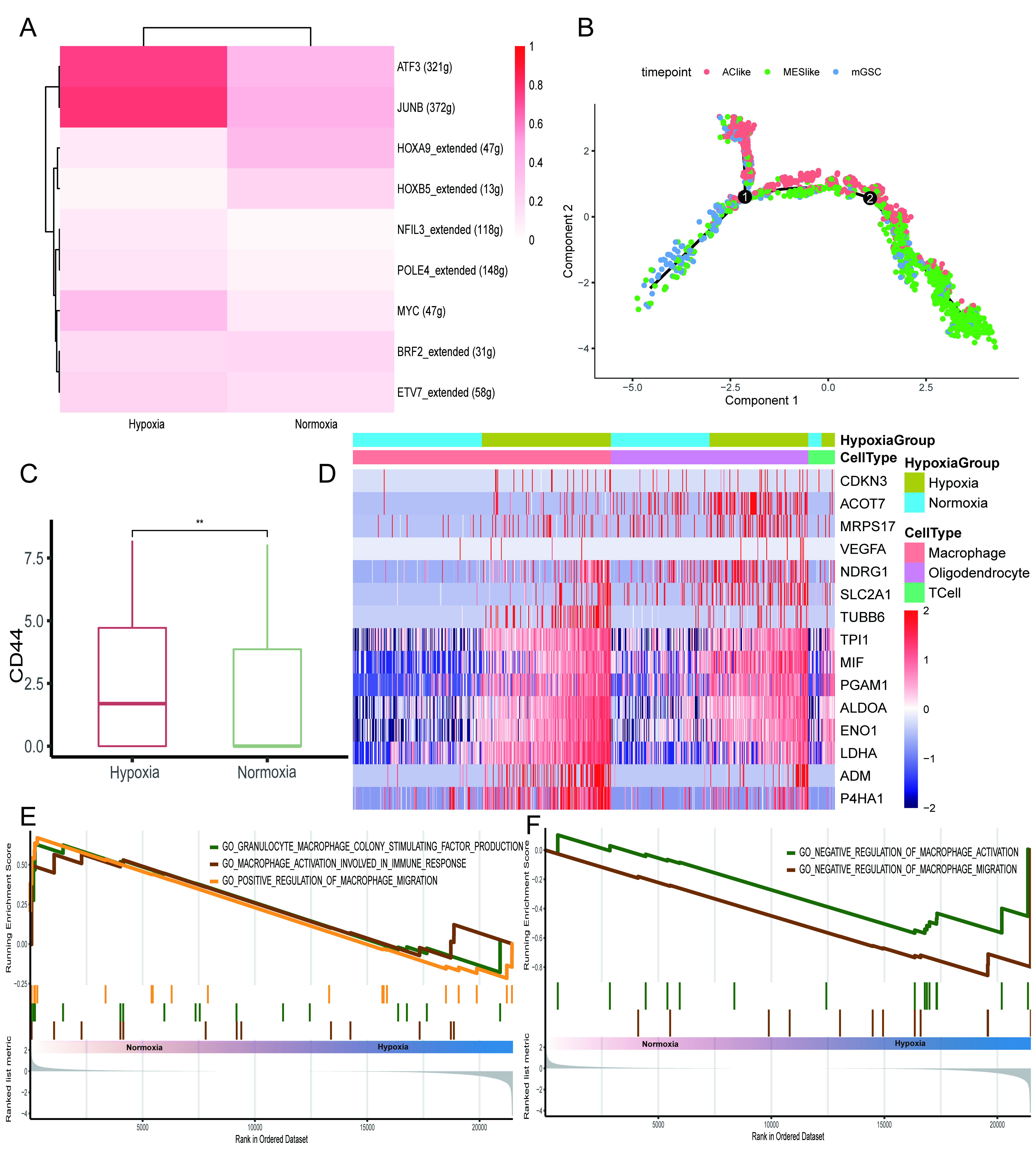

Supplement: Supplementary Figure 2 — (A) Heatmap of binarized regulon network activity in tumor cells with hypoxia or normoxia. (B) Lineage trajectory among mGSCs, MES-like cells and AC-like cells. (C) Distribution of CD44, MES marker, between hypoxia and normoxia groups. (D) The expression of 15 hypoxia-related genes in GBM non-tumor cells. (E) GSEA analysis plot of activated pathways in macrophage under normoxia condition. (F) GSEA analysis plot of activated pathways in macrophage under hypoxia condition. [file Image_2.tif]
